# Functional characterization of a new ORF βV1 encoded by radish leaf curl betasatellite

**DOI:** 10.3389/fpls.2022.972386

**Published:** 2022-09-20

**Authors:** Neha Gupta, Kishorekumar Reddy, Prabu Gnanasekaran, Ying Zhai, Supriya Chakraborty, Hanu R. Pappu

**Affiliations:** ^1^Molecular Virology Laboratory, School of Life Sciences, Jawaharlal Nehru University, New Delhi, India; ^2^Department of Plant Pathology, Washington State University, Pullman, WA, United States

**Keywords:** begomovirus, radish leaf curl betasatellite, hypersensitive response, βV1, reactive oxygen species, tomato leaf curl New Delhi virus, pathogenesis, replication enhancer protein

## Abstract

Whitefly-transmitted begomoviruses infect and damage a wide range of food, feed, and fiber crops worldwide. Some of these viruses are associated with betasatellite molecules that are known to enhance viral pathogenesis. In this study, we investigated the function of a novel βV1 protein encoded by radish leaf curl betasatellite (RaLCB) by overexpressing the protein using potato virus X (PVX)-based virus vector in *Nicotiana benthamiana*. βV1 protein induced lesions on leaves, suggestive of hypersensitive response (HR), indicating cell death. The HR reaction induced by βV1 protein was accompanied by an increased accumulation of reactive oxygen species (ROS), free radicals, and HR-related transcripts. Subcellular localization through confocal microscopy revealed that βV1 protein localizes to the cellular periphery. βV1 was also found to interact with replication enhancer protein (AC3) of helper virus in the nucleus. The current findings suggest that βV1 functions as a protein elicitor and a pathogenicity determinant.

## Introduction

Members of geminivirus group contain single-stranded circular DNA genome and infect diverse monocot and dicot plants, causing tremendous economic loss to global agronomy. Recent advances in metagenomics disclosed several novel geminiviruses from divergent hosts, leading to the expansion of geminivirus classification. Currently, the family *Geminiviridae* is composed of 14 genera based on the genomic composition, insect vector, host range, and phylogenetic analysis. They are *Becurtovirus*, *Begomovirus*, *Curtovirus*, *Mastrevirus*, *Capulavirus*, *Eragrovirus*, *Grablovirus*, *Topocuvirus*, *Turncurtovirus*, *Citlodavirus*, *Maldovirus*, *Mulcrilevirus*, *Opunvirus*, and *Topilevirus*. Whitefly-transmitted Begomoviruses represent the largest genus constituting 445 out of approximately 520 geminiviral species (Fiallo-Olivé et al., 2021).

Geminivirus encodes for proteins on both virion and complementary sense strands and transcription mediated by bidirectional promoters present in the conserved regions. The limited coding capacity of geminiviruses is overcome by efficient utilization of host transcription and translation machinery and hijacking of host cellular factors (Hanley-Bowdoin et al., 2013; Gupta et al., 2021). During the infection process, capsid uncoating followed by geminivirus gene expression enables the activation of both the early genes essential for replication and the suppressors of host defense responses. Rep protein encoded by DNA-A or helper begomovirus essentially plays a regulatory switch between rolling circle replication, and early and late transcription events. The expression of late genes facilitates the synthesis of nuclear shuttle protein (NSP) and movement proteins (MP) to support virus movement across the plant. Coat protein (CP) promotes insect transmission of disease complexes.

Depending on the genomic composition, begomovirus can be either bipartite (contains DNA-A and DNA-B genomes) or monopartite (contain only a single genome known as helper virus), which shares homology with DNA-A of the bipartite counterpart (Hanley-Bowdoin et al., 2000). Monopartite begomoviruses are often accompanied by betasatellites that are known to regulate pathogenesis (Cui et al., 2004; Briddon and Stanley, 2006). Betasatellites do not share sequence homology with their helper viruses, except at the satellite conserved region (SCR) indispensable for *trans*-replication by helper begomovirus. Betasatellite encoded βC1 protein conditions the cellular machinery to facilitate the survival and proliferation of begomovirus-betasatellite complexes. It also suppresses host defense, augments disease severity, and enhances the vector performance and it influences the viral pathogenesis through its novel ATP hydrolysis activity (Gnanasekaran et al., 2019, 2021).

Geminivirus gene expression *via* transcription regulation by bidirectional promoters has been well characterized across all the genera. Transcript mapping for multiple geminiviruses such as African cassava mosaic virus (ACMV), maize streak virus (MSV), tomato golden mosaic virus (TGMV), digitaria streak virus (DSV), cotton leaf curl burewala virus (CLCuBuV) and, mungbean yellow mosaic virus (MYMV) revealed the occurrence of polyadenylated transcripts and their regulation by bidirectional promoters (Morris-Krsinich et al., 1985; Townsend et al., 1985; Accotto et al., 1989; Sunter and Bisaro, 1989; Shivaprasad et al., 2005; Akbar et al., 2012). Two large polycistronic mRNAs from DNA-A can encode for all the complementary sense transcripts such as AC1, AC2, AC3, AC4, and AC5 through internal AUG regulated by ribosomal leaky scanning, whereas virion sense transcripts AV1 and AV2 are generated from the single transcript (Shivaprasad et al., 2005). Emerging studies have annotated additional V3 ORF from the genomes of *Becurtovirus*, *Curtovirus*, *Grablovirus*, and *Topilevirus*, as well as V3 and V4 ORFs from *Capulavirus*, *Citlodavirus*, and *Mulcrilevirus* (Fiallo-Olivé et al., 2021). Intriguingly, promoter activation assays of tomato yellow leaf curl virus (TYLCV) demonstrated the ability of geminivirus to express additional small proteins encoded by ORF 1, 2, 4, and 5 with potential virulence during the infection (Gong et al., 2021). DNA-B encodes for two canonical transcripts, BC1 and BV1, *via* bidirectional promoters embedded in the CR region, similar to DNA-A transcripts. Recent robust technical advancements uncovered several non-canonical proteins from DNA-A and DNA-B of tomato yellow leaf curl Thailand virus (TYLCTHV) that included protein isoforms of a previously well-defined AV2 protein and a novel BV2 protein with distinct subcellular localization and role in pathogenicity (Chiu et al., 2022). Across the geminivirus species, virus-encoded proteins are multifunctional. Although they are positional homologs between the genomic components, their functional diversity overrides host defense and facilitates successful virus infection in the host cell (Luna and Lozano-Durán, 2020; Devendran et al., 2022).

Betasatellites modulate helper virus replication, augment disease severity, suppress host defense, and enhance vector performance. Multiple sequences and mutagenic studies on betasatellite speculated the origin of different transcripts other than predominant βC1 transcripts. A 5.3 kDa functional protein overlapping with βC1 on virion sense strand has been predicted with TESTCODE, based on the codon usage of AYVV DNA betasatellite (Saunders et al., 2000). Similarly, V1, V2 and V3 transcripts were predicted approximately at the overlapping sequence of βC1 ORF in CLCuV DNA β (Briddon et al., 2001). Detection of βC1 by full-length AYYV DNA beta probe resulted in a smear kind pattern of up to 1.35 kb, which had not cleared even after two successive DNase I treatments, which raises the speculation of additional transcripts (Saunders et al., 2004). Mutagenic studies on βV1 ORF revealed no additive effect on symptom enhancement and pathogenesis and further could not be mapped through RACE (Saeed et al., 2005). However, the origin of additional transcripts, either coding or non-coding, cannot be ruled out as the emergence of betasatellite has been rapid. Also, to cope with the host defense machinery, betasatellite must have evolved unexplored alternative attacking strategies to gain control over the host, thus maintaining host-virus coevolution. Recently the functional βV1 ORF has been detected on the virion sense strand of tomato yellow leaf curl China betasatellite (TYLCCNB; Hu et al., 2020). It plays a vital role during viral infection and induces hypersensitive response (HR)-type cell death (Hu et al., 2020). Radish leaf curl betasatellite (RaLCB; Rβ) is associated with tomato leaf curl New Delhi virus (ToLCNDV; NA), thwarting host defense and aggravating disease symptoms in *N. benthamiana* (Bhattacharyya et al., 2015; Gnanasekaran et al., 2019). Deletion of sequence downstream to Rβ-βC1 from 56–187 caused a loss of the stem curling phenotype leading to the speculation of additional unidentified viral factors with potential pathogenicity functions (Reddy et al., 2020). In the current study, we identified novel transcripts originating from the Rβ and characterized a new βV1 ORF protein by *in silico* and molecular approaches.

## Materials and methods

### RNA extraction and northern blotting

*N. benthamiana* plants were inoculated with *Agrobacterium* strain EHA105 harboring helper virus NA and Rβ WT or Rβ mutant constructs, and total RNA was extracted from the systemic leaves subjected to DNase I treatment. Northern blotting was performed to identify the putative transcripts from Rβ during the virus infection. Total genomic RNA (10 μg) was electrophoresed on 1.2% denaturing formaldehyde agarose gel and transferred onto a nylon membrane for (Hybond-N+Amersham) overnight by a conventional capillary method. Then, transferred RNA was cross-linked to membrane using UVP Crosslinker (Amersham). SCR-DNA was radiolabelled with [α-32P]dCTP and hybridized to RNA blots. Later, it was scanned for radioactive signals using phosphorimager (Typhoon FLA 9500, GE Healthcare Life sciences, Illinois, United States).

### Random amplification of cDNA ends (RACE)

Single strand cDNA preparation and mapping of virion sense strand transcripts from SCR were carried out using SMARTer® RACE 5′/3′ Kit (Clontech Laboratories, Inc., California, United States) as per the manufacturer’s instructions. Nested PCR was performed using 5′ RACE gene-specific primer (GSP) and universal primer mix (UPM). Resultant PCR products were cloned into the pJET1.2 blunt-end vector and sequenced.

### Bioinformatics analysis

Transmembrane topology prediction and gene ontology were carried out using TOPCONS (Tsirigos et al., 2015) and the FFpred3 tool (Cozzetto et al., 2016). WoLF PSORT^1^ and Cell-PLoc2.0 (Chou and Shen, 2008) tools were used to predict signal peptide and subcellular localization of βV1 protein in the cellular environment.

### Plasmid construction

The βV1 ORF (351 bp) of Rβ was PCR amplified from an infectious clone of Rβ with the specific primer pair and cloned into a pJET1.2 vector at sites compatible with the vector. For overexpression studies *in planta*, the βV1 and βC1 ORFs were amplified using 106KV1FP/ 106KV1RP and RLBETA106FP/RLBETA106RP primer pairs (Supplementary Table S1) respectively, to obtain pJET1.2-βV1 and pJET1.2-βC1 clones. Then pGR106-βV1 and pGR106-βC1 constructs were generated by digesting pJET1.2 clone at *Cla*I and *Sal*I restriction sites and subsequently ligated with the pGR106 vector at the same sites. Similarly, the βV1 ORF was cloned into the pCAMBIA1302 vector for localization study at *Nco*I and *Spe*I restriction sites and confirmed using RK11301FP/ RK11301RP primer pair. The same strategy was used to clone βV1 and βC1 ORFs in pBinAR vector at *BamH*I and *Sal*I restriction site. For yeast two-hybrid assay (Y2H), the pGBKT7-βV1 construct was generated by first obtaining pJET1.2-βV1 clone using primer pair KV1KT7FP/106KV1RP. The construct pJET1.2-βV1 was digested and ligated to linearized pGBKT7 vector at *BamH*I and *Sal*I restriction site. ORFs of NA such as AC1, AC3, AV1, and AV3 were amplified from a monomeric NA DNA for cloning into pJET1.2 vector using specific primers (Supplementary Table S1) and further cloned in-frame into Y2H vector pGADT7 at compatible restriction sites. For Bimolecular fluorescence complementation (BiFC) studies, pSITE-EYFP-N1: βV1 and pSITE-EYFP-C1:AC3 constructs were generated using a gateway based cloning strategy. All the clones were ascertained by sequencing. Further, the clones for *in planta* expression were transformed into specific *Agrobacterium* strains.

### Agroinoculation and PVX-based expression *in planta*

*Agrobacterium*-mediated virus infiltration was performed according to the method suggested (Lee and Yang, 2006). *Agrobacterium tumefaciens* strain GV3101+ pJIC Sa_Rep harboring pGR106 vector alone, pGR106-βV1 and pGR106-βC1 were cultured in Luria-Bertani (LB) medium containing rifampicin (30 μg/ml) and kanamycin (50 μg/ml) at 28°C for 36–48 h at 200 rpm. The culture was centrifuged and resuspended in an agroinfiltration buffer consisting of 10 mM MES buffer (pH 5.8), 10 mM MgCl_2_, and 100 μM acetosyringone. Optical density (OD) was set to 0.5 at a wavelength of 600 nm, and the inoculum was incubated in the dark for 3 h. Dark-adapted (2–3 h), 3-week-old *N. benthamiana* plants were infiltrated through the abaxial side of young, lower leaves with the aid of a 1 ml needleless syringe. Similarly, pBinAR-βV1 and pBinAR-βC1, and pBinAR vector alone were infiltrated into the *N. benthamiana* plants.

### 3,3-Diaminobenzidine (DAB) staining

Detection of hydrogen peroxide (H_2_O_2_) was done using the DAB-uptake method as per (Orozco-Cardenas and Ryan, 1999; Daudi and O’Brien, 2012) with slight modifications. βV1 and βC1 overexpressing constructs were infiltrated in leaves of 3-week-old *N.benthamiana* plants. At 48 hpi, 5 dpi, and 7 dpi, those leaves were excised from the petiole and immersed in a petri dish filled with freshly prepared 1 mg/ml DAB-HCl solution. For 100 ml DAB solution, 100 mg of DAB (Sigma-Aldrich) was added in 90 ml of sterile water and dissolved using a magnetic stirrer. The pH of the solution was reduced to 3.0 using 0.2 N HCl. Since DAB is light-sensitive, cover the container with aluminum foil. 50 μl of Tween 20 (0.05% v/v) and 5 ml of 200 mM Na_2_HPO_4_ were added to the solution, and final volume was made up to 100 ml. Place the covered petri dishes on a laboratory shaker at 200 rpm for 10 h. After incubation, the leaves were placed in absolute ethanol overnight to bleach out the chlorophyll, and photography was done.

### Nitro blue tetrazolium chloride (NBT) staining

For detection of superoxide (O_2_^−^), 0.2 gm NBT stain (Sigma Aldrich) was dissolved in 100 ml of 50 mM sodium phosphate buffer (pH 7.5) to make a 0.2% NBT solution. *N.benthamiana* leaves infiltrated with βV1 and βC1 overexpressing constructs were collected at 48 hpi, 5 dpi, and 7dpi and placed immediately into the NBT solution, and incubated on a laboratory shaker at lower rpm for 10 h for stain uptake. Later, to bleach out the chlorophyll, the leaves were immersed in absolute ethanol overnight and photographed.

### Trypan blue staining

Tissue cell death was visualized using a trypan blue stain. The stain solution was prepared by dissolving lactic acid (85% w,w): phenol (TE-saturated pH 7.5–8.0): glycerol: H_2_O in the ratio of 1:1:1:1 along with 100 mg trypan blue stain for 100 ml. Infiltrated leaves were excised from petiole and immersed in trypan blue stain for 10 h. The leaves were then transferred to absolute ethanol for overnight, and photography was carried out.

### Quantitative real-time PCR (qRT-PCR) and analysis

qRT-PCR was performed using SSoAdvanced Universal SYBR Green Supermix (Biorad). In a reaction volume of 20 μl, 10 μl of 2× SYBR Green Supermix was added to 2 μl of 50 ng cDNA, 0.7 μl of 10 μM of forward and reverse primer and remaining 6.6 μl of nuclease-free water. The Applied Biosystems 7,500 fast real-time PCR was programmed for DNA denaturation at 95°C for 30 s, repeating 95°C for 15 s (40 cycles), and finally, annealing and extension at 60°C for 30 s. The reference control gene used for normalization was protein phosphatase 2A (PP2A). Relative gene expression was determined by the 2^-ΔΔCt^ method (Schmittgen and Livak, 2008). The experiment was performed using three biological replicates and two technical replicates. Statistical analysis was carried out using one way ANOVA that identified significant difference between the samples, with significant (*p* < 0.05), highly significant (*p* < 0.01) and very highly significant (*p* < 0.001).

### Confocal microscopy

*A. tumefaciens* strain GV3101 harboring pCAMBIA1302-βV1 construct was infiltrated into three-weeks old *N. benthamiana* plants for subcellular localization study, while for BiFC, pSITE-EYFP-N1: βV1 and pSITE-EYFP-C1:AC3 constructs were used. After 48 h post-infiltration, the epidermal cells of infiltrated leaves were visualized under TCS SP8 X confocal microscope (Leica, Germany), and images were acquired. For nuclear detection, 4′,6-diamidino-2-phenylindole (DAPI) staining was used. The excitation and emission filter used for DAPI fluorescence were 340–380 nm and 435–485 nm, respectively.

Yellow fluorescent protein (YFP) fluorescence was observed at 514 nm excitation and 520 nm-540 nm emission. For green fluorescent protein (GFP) signal, excitation, and emission were at 465–495 nm and 515–555 nm, respectively.

### Yeast two-hybrid analysis (Y2H)

Y2H assay was carried out by expressing βV1 protein in fusion to GAL4 DNA-binding domain (pGBKT7-βV1) and viral proteins fused to GAL4 activation domain (pGADT7-AC1, pGADT7-AC2, pGADT7-AC3, pGADT7-AC4, pGADT7-AV1, pGADT7-AV2, and pGADT7-AV3) in AH109 strain of yeast. AH109 yeast strain was grown till OD reached 0.5. The yeast culture was pelletized at 3,000 rpm for 5 min at room temperature and treated with 10X LiAc buffer. The appropriate plasmid combinations were co-transformed into the yeast culture. The transformed colonies were grown on a synthetic defined (SD) double dropout media. Further, the interaction was confirmed by growing transformants on selection media (SD)-Leu-Trp-His having 2 mM 3-amino-1,2,4-triazole (3AT).

## Results

### Identification of radish leaf curl betasatellite encoded novel transcripts

**Figure 1:**
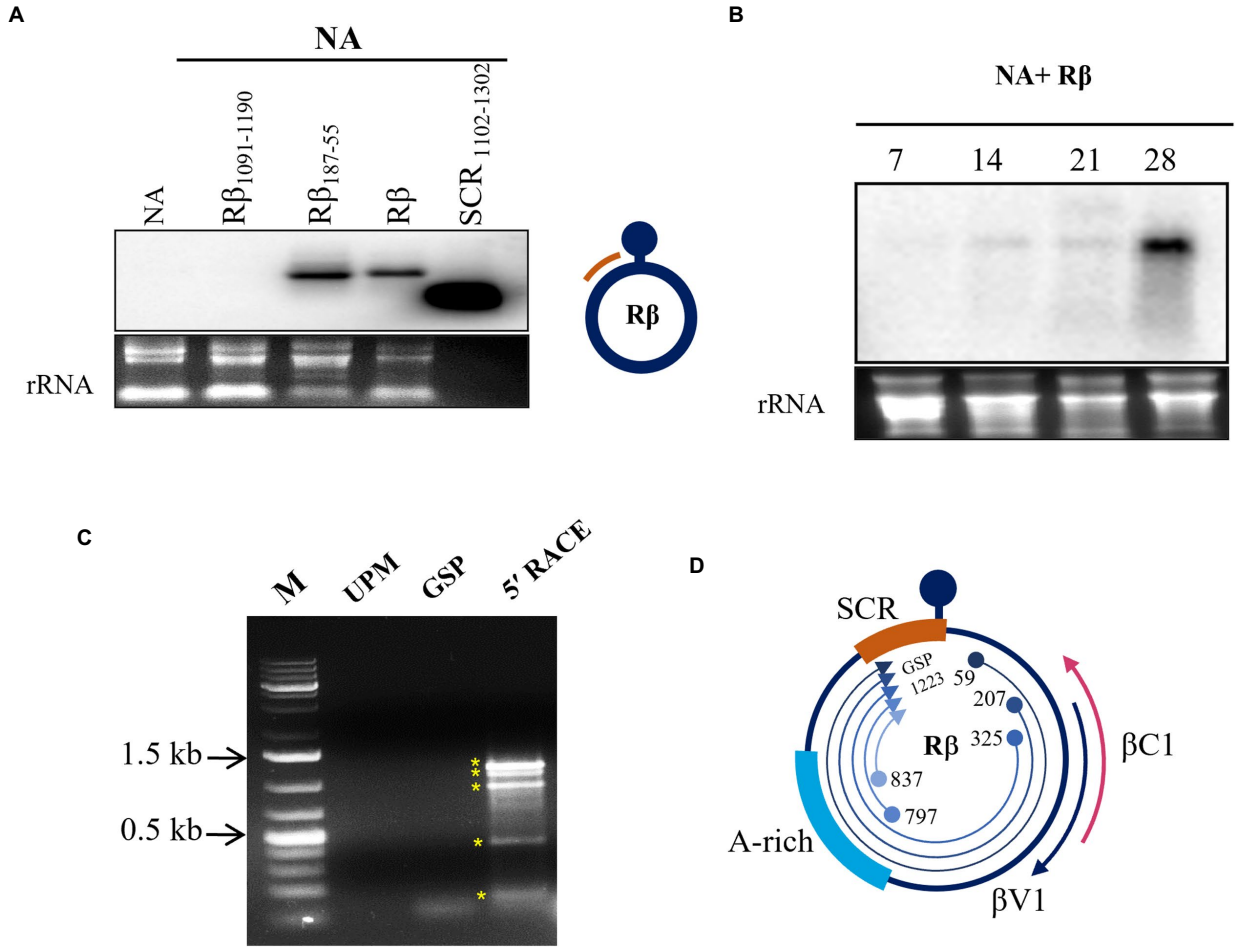
Transcript detection of Rβ specific transcripts from NA+ Rβ infected *N. benthamiana* plants. Total RNA extracted from NA alone and NA with Rβ_1091-1190,_ Rβ_187-55_ and Rβ inoculated plants at 19 dpi used for hybridization with SCR specific radiolabelled probe. **(A)** Hybridization signals are detected in Rβ_187-55_ and Rβ. The DNA used for probing served as positive control and the location of probe from Rβ indicated in orange color. **(B)** Temporal expression of SCR specific transcripts was detected during the disease progression at 7, 14, 21, and 28 dpi. Ethidium bromide-stained ribosomal RNA (rRNA) used as a loading control. **(C)** 5′ RACE nested PCR of virion sense SCR transcript mapping. M indicates DNA marker, UPM and GSP stands for universal primer mix and gene specific primer, respectively, which served as a negative control for 5′ RACE and nested PCR amplicons, indicated with asterisks (*). **(D)** Schematic presentation of Rβ and overview of SCR originated 5′ RACE transcript mapping. Location, orientation of TSS and GSP binding site are indicated.

SCR of Rβ is composed of key elements essential for betasatellite replication and maintenance. Our previous study of Rβ mutant inoculation studies in *N. benthamiana* indicated that the deletion of sequences proximity to SCR leads to alterations in betasatellite mediated symptoms raising the speculation of hidden pathogenicity factors (Reddy et al., 2020). To identify the novel transcripts originated from SCR during NA+ Rβ infection, virus constructs of NA alone or NA with wild type Rβ, Rβ_1091-1190_ (deleted for a part of SCR), and Rβ_187-55_ (deleted for a sequence region downstream to βC1 ORF) were inoculated in *N. benthamiana* plants and total RNA isolated at 19 dpi was subjected to northern blotting using an SCR specific radiolabeled probe. Intriguingly, a transcript signal was identified in wild-type Rβ and Rβ_187-55_ plants but not in the NA alone or NA+ Rβ_1091-1190,_ indicating that the SCR region potentially contains the transcript origins (Figure 1A). Further, the SCR-transcript accumulation was monitored at 7, 14, 21, and 28 dpi for temporal expression from early to later days of NA+Rβ infection (Figure 1B). As the disease progresses, SCR-transcripts tend to have a gradual increased levels of transcript accumulation with multiple fold increase from 21 to 28 dpi, indicating a possible functional role during the late phase of the infection. To gain insights into putative transcript origins, 5′ RACE was carried out for SCR by sequence-specific nested PCR, and the resultant PCR amplicons were purified, cloned, and analyzed by Sanger sequencing. Virion sense 5′ RACE resulted in five different amplification products with transcriptional start sites (TSS) at 59, 207, 325, 797, and 837 of Rβ, while the gene-specific primers bound at 1223 of SCR (Figures 1C, D). These multiple transcripts could be five individual transcripts, derivatives or truncated 5′ terminus products of larger transcript units. Further, these sequences were analyzed by an ORF finder to find out the novel protein-coding regions of a minimum of 75 nucleotides (nt) to locate the potential protein-coding ORFs. At least five ORFs were found in the range of 78 to 351 nt on the virion sense strand. The transcript of 351 nt has caught our attention as it falls downstream to the region that is responsible for stem curling and has been found with Fangorn Forest (F2) method that classifies geminivirus genes based on the machine learning approach (Silva et al., 2017) and SnapGene viewer. To gain insights into the molecular function of βV1, gene ontology (GO) of βV1 was analyzed by the FFPred3 tool and prediction results are sorted based on the high probability and reliability of Support Vector Machines (SVM), categorized into biological processes, molecular functions, and cellular components predictions. Presumably, βV1 contains nucleoside or nucleotide-binding activity and may associate with membrane transport function (Supplementary Table S2).

### Overexpression of βV1 induces hypersensitive response

**Figure 2:**
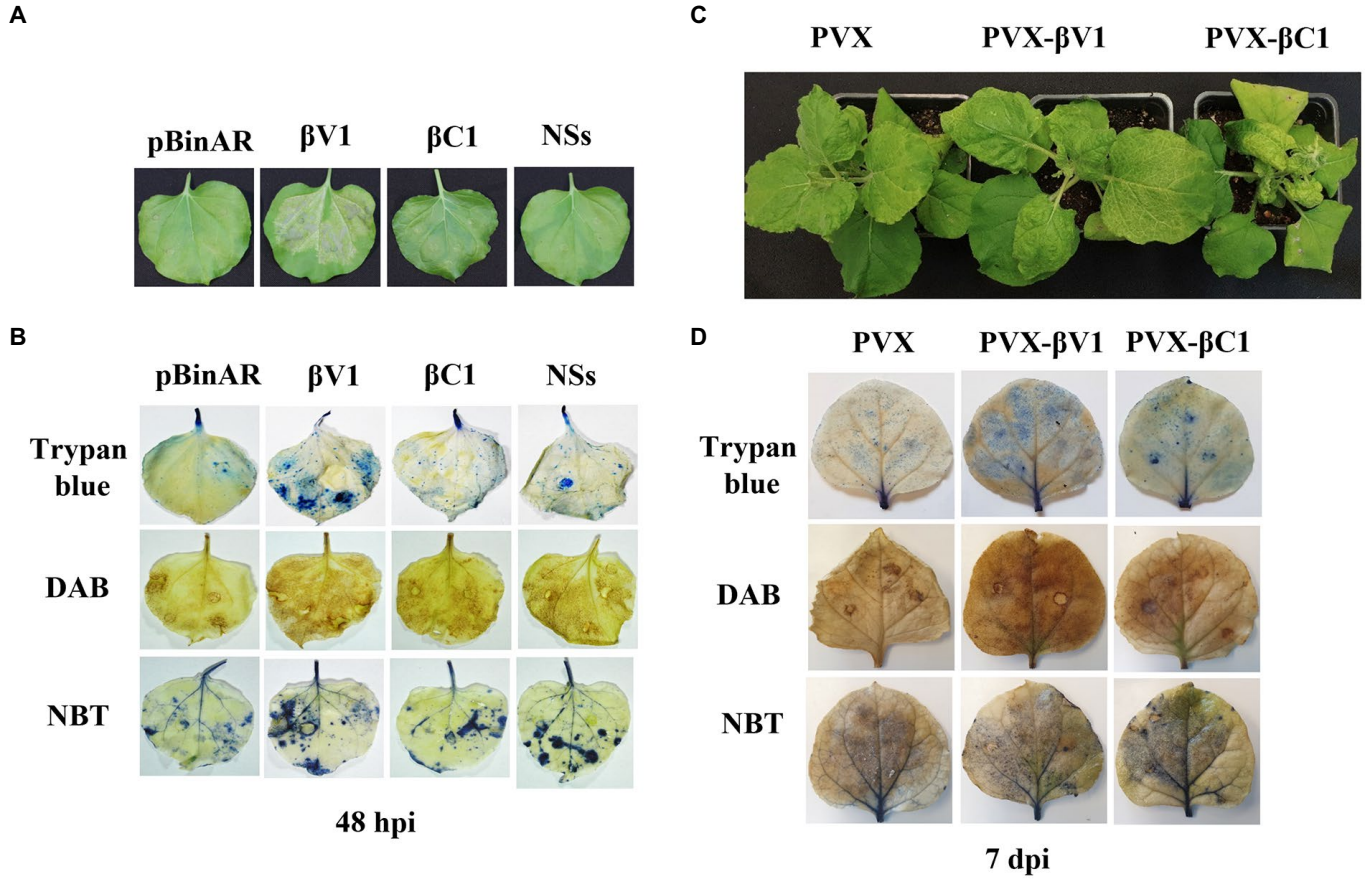
βV1 protein acts as inducer of HR response. **(A)** Cell death observed after 48 h post infiltration of pBinAR-βV1 and pBinAR-βC1 constructs in *N. benthamiana*. **(B)** Photographed infected leaves stained with DAB, NBT, and trypan blue showing accumulation of ROS generation and associated with cell death. **(C)** Symptoms on *N. benthamiana* plants infected with PVX, PVX-βV1, and PVX-βC1 at 10 dpi. **(D)** Photographed PVX constructs infiltrated leaf pictures stained with DAB, NBT, and trypan blue at 7 dpi.

To understand the role of βV1 in Rβ mediated pathogenicity and perception by the plant immune system, Rβ-βV1 was cloned under the control of *CaMV* 35S promoter and transiently expressed in *N. benthamiana via* agroinfiltration. Infiltrated leaves exhibited HR-like lesions after 48 h post-infiltration at the site of the infiltration patch (Figure 2A). Plants infiltrated with empty vector (pBinAR) did not show visible lesions. NSs protein of groundnut bud necrosis virus (GBNV), previously reported to induce cell death, was used as a positive control (Singh et al., 2017). Intriguingly, Rβ-βC1/35S also caused HR-like necrosis. The HR response is one of the earliest defense responses of the plant towards pathogen prior to cell death. The initial recognition of the avirulence factor or elicitor causes oxidative burst, accumulating reactive oxygen species (ROS), that leads to cellular damage. Therefore, HR is usually preceded by ROS and free radicals accumulation in the cells. To investigate the presence of hydrogen peroxides (H_2_O_2_) and superoxides anions (O_2_-), leaves infiltrated with βV1/35S, βC1/35S, and control were subjected to DAB and NBT staining. βV1/35S and βC1/35S infiltrated leaves displayed the accumulation of insoluble brown and blue precipitate, respectively, that confirms the ROS accumulation (Figure 2B). HR-induced cell death was further confirmed by trypan blue staining of agroinfiltrated leaves. βV1 and βC1 overexpressed leaf tissue got distinct blue color stain indicating cell death (Figure 2B). The accumulation of H_2_O_2_ was higher in βV1and βC1 expressed leaves compared to the pBinAR vector (Figure 2B). βV1 and βC1 proteins were also expressed ectopically through *Potato virus X* (PVX). In plants infiltrated with PVX alone (pGR106 vector), very mild mosaic symptoms appeared on the systemic leaves at around 7 dpi (Figure 2C). However, PVX-βV1 expressed plants showed severe necrosis at the systemic leaves, especially at veins, and symptoms like mild leaf curling, enations, chlorosis, and mosaic symptoms. This indicates that βV1 acts as a pathogenicity determinant. The necrotic lesions were also observed at local tissue resembling the hypersensitive response (HR) type cell death. PVX-βC1 expressing plants displayed typical betasatellite-associated symptoms like leaf curling, stem bending, and stunted growth (Figure 2C). Additionally, the infiltrated leaves were stained with DAB, NBT, and trypan blue at 5 dpi and 7 dpi. As expected, PVX-βV1 showed higher ROS and free radical accumulation than the PVX-βC1 and PVX alone, which further supports the role of βV1 as an inducer of cell death. βV1 induced cell death was also supported by staining of PVX-βV1 infiltrated plants with trypan blue (Figure 2D; Supplementary Figure S1).

### Overexpression of βV1 and βC1 alters the expression of hypersensitive response and pathogenesis-related transcripts

**Figure 3:**
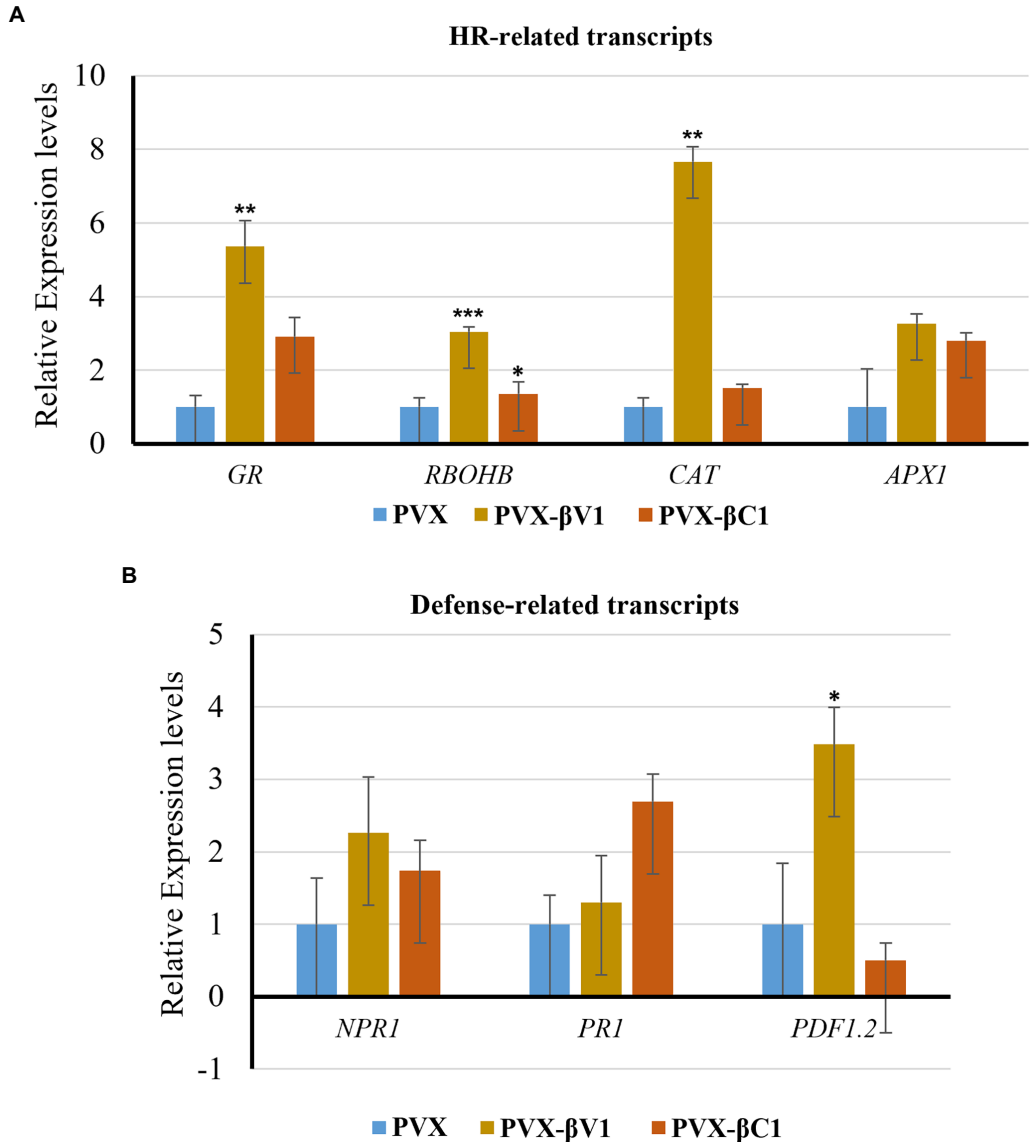
Transcript analysis through qRT-PCR. Relative expression levels of **(A)** HR-related transcripts (*RBOHB*, *CAT*, *GR*, and *APX1*), and **(B)** defense-related transcripts (*NPR1*, *PR1*, and *PDF1.2*) in *N. benthamiana* leaves infiltrated with PVX-βV1 and PVX-βC1 at 5 dpi. The *PP2A* gene was used as a reference control to normalize the transcripts. The error bar represents the standard error, and the asterisk on the top of the bars indicates a statistically significant difference.

As the overexpression of βV1 resulted into ROS generation, we detected the expression level of HR-related and defense-related genes in both βV1 and βC1 overexpressing plants. Many transcripts regulating ROS homeostasis like respiratory burst oxidase homologues B (*RBOHB*), ascorbate peroxidase 1 (*APX1*), glutathione reductase (*GR*), Catalase (*CAT*), and pathogenesis-related transcripts like the non-expressor of pathogenesis-related 1 (*NPR1*), pathogenesis-related protein 1 (*PR1*) and plant defensin 1.2 (*PDF1.2*) were selected (Maleck and Dietrich, 1999; Guidetti-Gonzalez et al., 2007; Das and Roychoudhury, 2014). *N. benthamiana* plants were agroinfiltrated with either PVX-βV1, PVX-βC1 overexpression constructs or PVX alone. Total RNA was isolated for the expression analysis at 5 dpi and qRT-PCR was carried out (Figure 2C). The reference gene used for normalization was protein phosphatase 2A (*PP2A*). *RBOHB*, which plays a major role in hydrogen peroxide production in the cells, increased to 2.7-fold and 1.3-fold in PVX-βV1 and PVX-βC1, respectively (Figure 3A). Interestingly, the transcript level of *GR*, *CAT*, and *APX1* (ROS scavenging enzymes) which protects cells from the damaging impact of H_2_O_2_ and other free radicals were also upregulated in both PVX-βV1 and PVX-βC1 compared to PVX alone (Figure 3A). In leaves expressing PVX-βV1, the expression of *GR*, *CAT*, and *APX1* were increased to 6-, 6.6-, and 3.27-fold while expression in PVX-βC1 infected leaves was 2.7-, 1.5-, and 2.8-fold, respectively (Figure 3A). These results showed that the oxidative burst in cells during βV1 overexpression is accompanied by change in the transcript level of both ROS production and detoxification enzymes to check the action of expressed pathogenicity determinant. However, there is no significant increase in the expression of defense-related transcripts *NPR1* and *PR1* that regulates systemic acquired resistance triggered by HR response during pathogen attack. Interestingly, *PDF1.2* transcript was significantly upregulated in PVX-βV1 infiltrated plants (Figure 3B).

### βV1 protein encoded by RaLCB interacts with NA encoded AC3 protein in the nucleus

**Figure 4:**
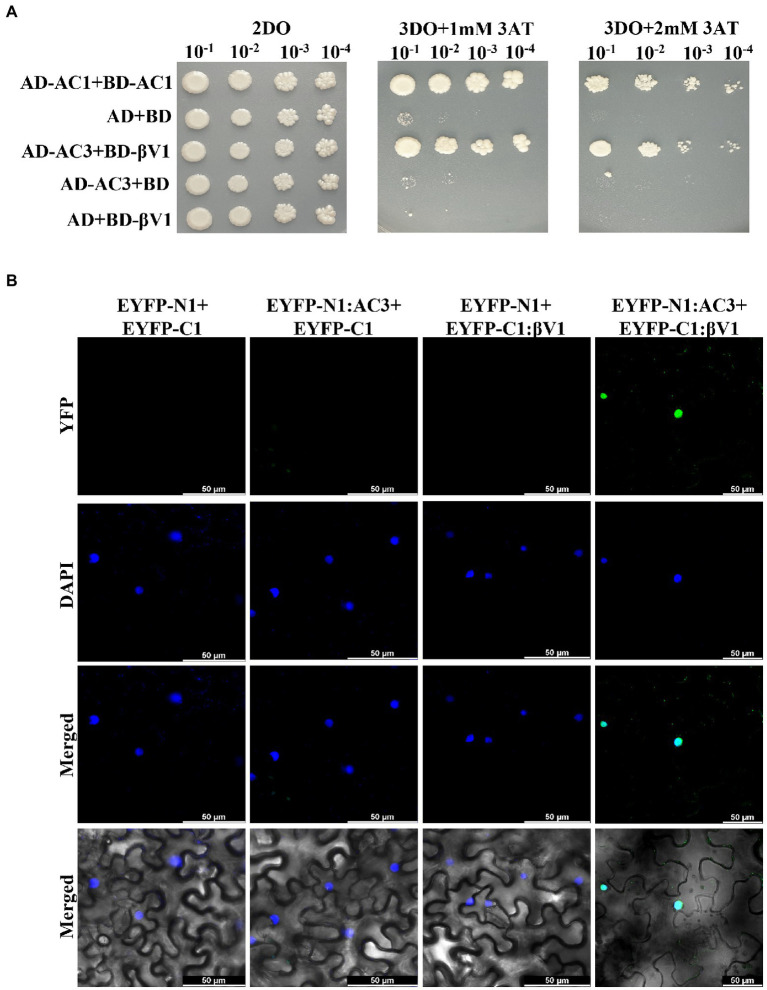
βV1 interacts with the AC3. **(A)** The yeast strain AH109 cells were co-transformed with different combinations of plasmids expressing AD-AC1+BD-AC1, AD+BD, AD-629 AC3+BD-βV1, AD-AC3+BD, or AD+BD-βV1. The co-transformed cells were grown on synthetic dropout media (2DO) or selection medium SD-Leu-Trp-His supplemented with either 1 mM or 2 mM 3-amino-1,2,4-triazole after serial dilutions from cultures of OD 1.0 at 600 nm. AD-AC1+BD-AC1 combination served as a positive control, while AD+BD combination acted as negative control. **(B)** *Agrobacterium* strain GV3101 harboring different combinations of BiFC constructs were co-infiltrated into leaves of *benthamiana* plants. After 48 h post infiltration, epidermal cells of leaves were visualized by confocal microscopy to detect the reconstituted YFP fluorescence. The nucleus was stained with DAPI. Row 1 shows YFP fluorescence. Row 2 shows DAPI fluorescence, while row 3 and 4 represents the merged images without and with DIC images, respectively. Scale bars represents 50 μm.

NA DNA-A association with RaLCB produces a severe array of symptoms in *N. benthamiana* plants. The dependence of the helper virus on betasatellite for intense disease development suggests an intricate network of connections between the betasatellite proteins and viral proteins. After functional characterization of the βV1 protein at an individualistic level, it is important to unveil this connection. To study the interaction between βV1 and NA encoded ORFs AC1 (Replication associated protein), AC2 (Transcription activator protein), AC3 (Replication enhancer protein), AC4, AV1 (Coat protein), AV2 (Pre-coat protein), and AV3, all DNA-A ORFs were cloned into yeast-two hybrid (Y2H) vector pGADT7 (represented as AD) and βV1 in pGBKT7 (represented as BD). Y2H assay was conducted by co-transforming AH109 yeast strain with desired combinations. Interestingly, yeast cells which were co-transformed with AD-AC3 and BD-βV1 were grown on selection media SD-Leu-Trp-His supplemented with 2 mM 3AT (Figure 4A). We did not detect any interaction between other viral proteins and βV1. The yeast transformants carrying AD-AC1 and BD-AC1 constructs were taken as a positive control, while a plasmid combination AD and BD was used for negative control. The interaction between AC3 and βV1 proteins was further validated *in planta* by BiFC assay. The three-week old *N. benthamiana* plants were co-infiltrated with pSITE-EYFP-N1: βV1 and pSITE-EYFP-C1:AC3 and the epidermal leaf sections were analyzed by confocal microscopy after 48 h. The reconstituted yellow fluorescent protein signal was observed in the nucleus, suggesting that AC3 and βV1 interacts in the nucleus (Figure 4B). The nuclei of epidermal leaf sections were detected by DAPI. No fluorescence was detected in leaves infiltrated with either EYFP-N1+EYFP-C1, EYFP-N1: βV1+EYFP-C1, orEYFP-N1+EYFP-C1:AC3 combinations.

### βV1 protein localizes to the cytoplasmic periphery

**Figure 5:**
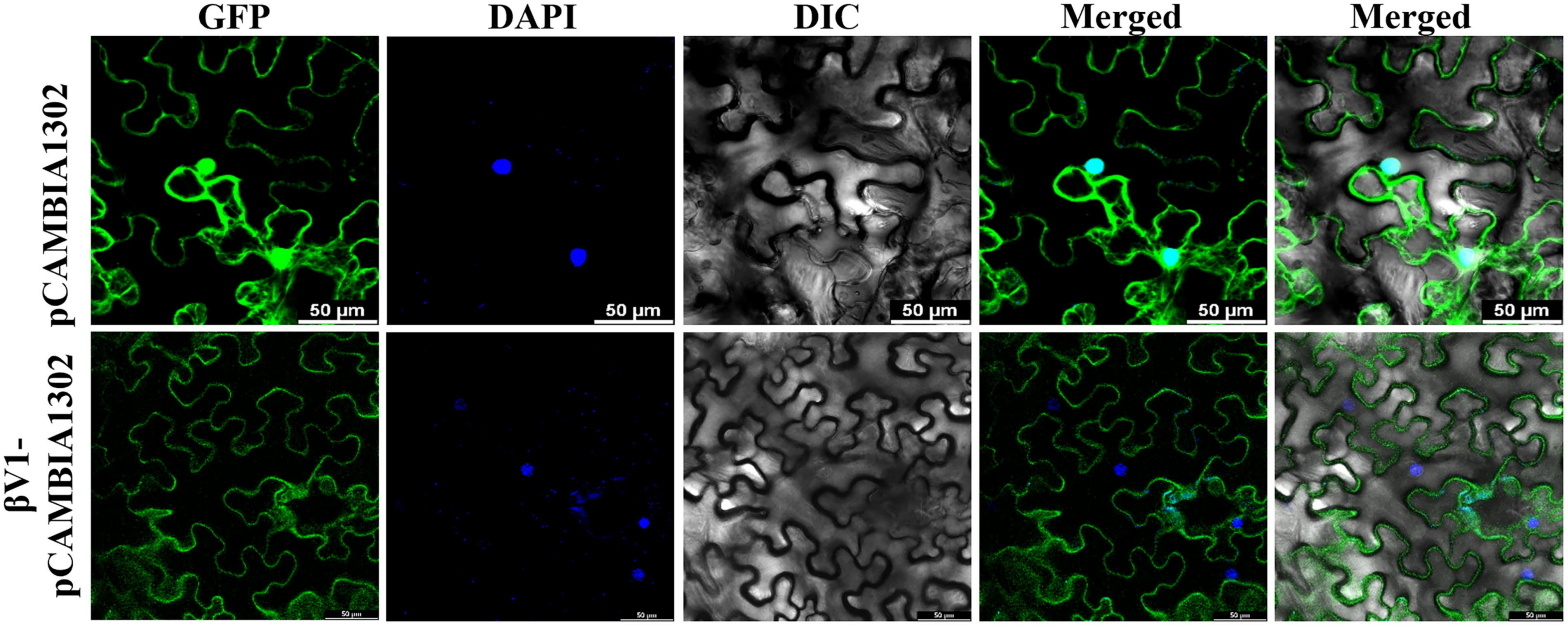
Localization of βV1 protein. The *N. benthamiana* leaves were agroinfiltrated either with pCAMBIA1302 or βV1-pCAMBIA1302. The epidermal cells were observed under confocal microscopy at 48 h post infiltration. Columns 1–3 show localization of GFP (upper panel) or βV1-GFP (lower panel), a nucleus stained with DAPI, DIC image of *N. benthamiana* epidermal cells. Columns 4 and 5 shows merged images without and with DIC images. The scale bar represents 50 μm.

The interaction of βV1 protein with AC3 protein the nucleus prompted us to study the intracellular localization of βV1 protein. To determine the sub-cellular localization of βV1 protein in epidermal cells of *N. benthamiana*, βV1 protein was fused to the N-terminus of green fluorescent protein (GFP) in plasmid pCAMBIA1302. The construct expressing fusion βV1-GFP protein was agroinfiltrated in the plants and infiltrated leaf sections were analyzed through confocal microscopy after 48 h. The result revealed the presence of βV1 protein in the cellular periphery. GFP signal was observed throughout the cell in the case of pCAMBIA1302 vector infiltrated samples. However, GFP fluorescence in βV1-pCAMBIA1302 infiltrated plants was present only at the cellular periphery (Figure 5). Additionally, the topology profile produced by the TOPCONS server indicates that βV1 could be a native transmembrane protein and contains a single transmembrane domain spanning from 94 to 114 amino acids, N-terminus 1–93 amino acids oriented towards the cytoplasm, and a short stretch of two amino acids (115–116) as a C-terminus non-cytoplasmic tail (Supplementary Figure S2). Although not validated here experimentally, but protein localization servers such as WoLF PSORT, Euk-mPLoc 2.0, BaCeILo, and plant-mPLoc also predicted that βV1 protein might be targeted to the plasma membrane or organellar membrane.

### Interaction with the AC3 proteins affects the localization of βV1 protein

**Figure 6:**
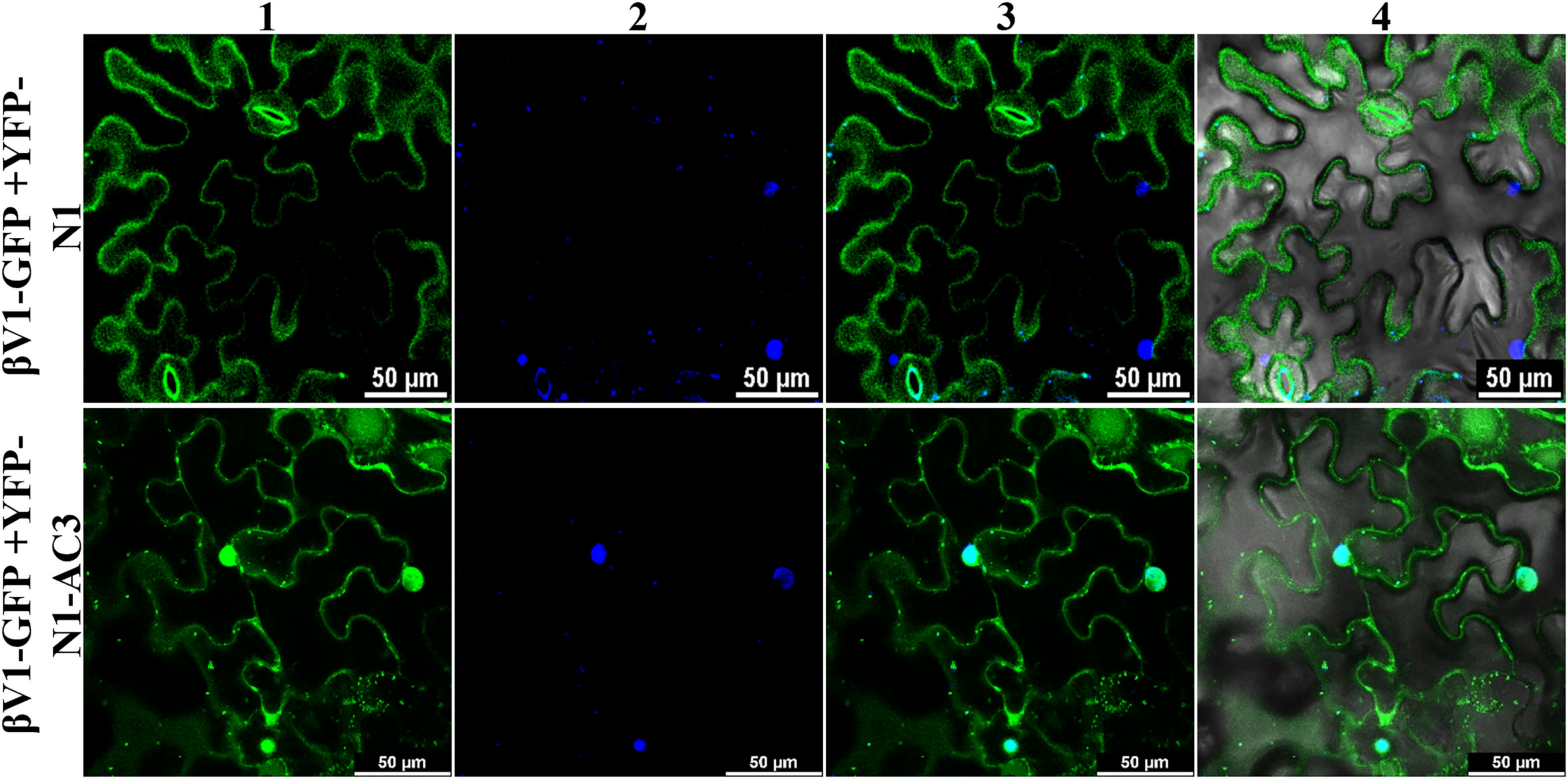
Co-expression of βV1-GFP and YFP-N1-AC3 fusion proteins. *N. benthamiana* leaves were agroinfiltrated with either βV1-GFP+YFP-N1 (upper row) or βV1-GFP+YFP-N1-AC3 (lower row). Epidermal cells were visualized under confocal microscopy at 48 h post infiltration. Column 1 represents the localization of βV1-GFP in the presence and absence of AC3 fusion protein. Column 2 indicates the nucleus stained with DAPI, while columns 3 and 4 represent merged images. Scale bars are equal to 50 μm.

To explore the ability of AC3 protein to alter the βV1 localization, βV1-GFP fusion protein was co-expressed with YFP-N1-AC3 fusion protein *via* agroinfiltration in *N. benthamiana* plants. The leaf sections observed by confocal microscopy after 48 h revealed that in the presence of AC3 protein, βV1 no longer showed distribution at cellular periphery, instead displayed nuclear sub-cellular localization, suggesting AC3 recruits βV1 from the cell periphery to the nucleus for interaction (Figure 6). Co-expression with AC3 alone is sufficient to bring about this shift in localization.

## Discussion

Viruses, because of their limited coding capacities, modulate the host cellular system by reprogramming the proteome and transcriptome of the host in a direction that proliferates the virus and enhances its pathogenesis. Association of betasatellite with begomovirus enhances symptom development, defense regulation, and movement. Until recently, betasatellite was known to encode a single protein βC1 of 13 kDa, in the complementary sense strand, which plays a key role in disease establishment. However, several pieces of evidence of host counter-responses highlight conquering the dominance of begomovirus disease complexes. These circumstances might drive the adaptation of novel genes to favor the geminiviruses and betasatellites coevolution. Newly identified small protein V3 encoded by TYCLV localizes to Golgi- and Endoplasmic reticulum. Interestingly, it is found to promote cell-to-cell movement of virus and functions as a suppressor of RNA silencing (Gong et al., 2022). Likewise, a new pathogenicity determinant C5 has been tracked down in TYLCV, that upon PVX-based expression, induces severe mosaic symptoms, increased viral accumulation, and ROS generation (Zhao et al., 2022). Considering the several crop losses caused by the begomovirus, identifying, and elucidating molecular mechanisms of pathogenicity determinants is one of the crucial measures for controlling the begomovirus diseases. In the current study, virion sense transcript mapping of SCR discloses the production of single or multiple transcripts from Rβ. Nevertheless, the experiments conducted to detect the strand-specific transcripts using virion or complementary sense probes by northern blotting failed. But a transcript was detected using a double-stranded radiolabeled DNA as a probe for northern blot, speculating that the multiple bands found during the 5′ RACE perhaps represent truncated transcripts of a single unique transcript. Although the bidirectional promoters have not been identified for betasatellite until now, it would not be a surprise as they are positionally conserved across the family *Geminiviridae* (Townsend et al., 1985; Accotto et al., 1989). With currently available information this is the first report showing the emergence of novel transcripts from the SCR of betasatellite.

Here, we characterized the function of βV1 protein, a novel protein encoded by the virion sense strand of Rβ. βV1 protein, when transiently expressed in *N. benthamiana*, was able to produce typical leaf curling symptoms. Moreover, βV1 was found to induce HR-type cell death which is usually not observed during geminiviral-betasatellite infection (Figure 2). HR is a form of resistance response generated in the plant during the incompatible interaction of the resistance gene (R-gene) in the host and a pathogen-associated avirulence gene (Avr gene) during the infection (Dangl and Jones, 2001). The results indicates that βV1 is a pathogenicity determinant and may acts as a protein elicitor or avirulence factor. During natural geminiviral infection, the viral molecular network may regulate the expression of βV1 towards the lower level to bypass the HR-related defense response for successful pathogenesis. βV1 induced ROS and free radicals generation have been detected by DAB and NBT staining. Cell death induced by HR was confirmed by trypan blue staining (Figure 2D). Additionally, the perturbation of HR and defense-related transcripts provided solid evidence to categorize βV1 as a pathogenicity determinant. Similar evidence of avirulence function was previously uncovered for C2 protein of tomato yellow leaf curl Sardinia virus, nuclear shuttle protein (NSP) of bean dwarf mosaic virus (BDMV) and V2 protein of tomato leaf curl Java virus-A (Zhou et al., 2007; Sharma and Ikegami, 2010; Matić et al., 2016). Similar HR induction was observed when the N-protein of tomato spotted wilt virus (TSWV) was over-expressed in *Capsicum chinense* and reported as an avr component of HR (Lovato et al., 2008). Many viral proteins like CI, P1, P3N-PIPO encoded by potyviruses, citrus tristeza virus encoding triplet proteins p33, p18, and p13 act as viral elicitors and pathogenicity determinants (García & Pallás, 2015).

Viral protein expands its functionality by interacting with other viral proteins or host proteins. The present study identified that βV1 protein interacts with the AC3 encoded by the helper virus NA (Figure 4). This interaction is observed in the nucleus. AC3 protein is known to localize into the nucleus and plays crucial role in enhancing the replication of viral DNA by rolling circle replication in the nucleus. It interacts with both proliferating cell nuclear antigen (PCNA) and rep protein and forms a replication complex (Castillo et al., 2003; Pasumarthy et al., 2011). However, the subcellular localization of βV1 through confocal microscopy reveals that βV1 protein localizes to the cellular periphery (Figure 5). Many viral proteins show dynamic sub-cellular localizations for successful pathogenesis (Li et al., 2020). For interaction, the AC3 protein possibly recruits βV1 from the cellular periphery into the nucleus since co-expression of both proteins brings the βV1 into the nucleus (Figure 6). The association of βV1 with AC3 protein may regulate viral replication and pathogenesis owing to the role of AC3 in enhancing virus replication. βV1 protein is also predicted to have a single transmembrane signal with membranous localization and transporter/cation membrane transport function (Supplementary Figure S2; Supplementary Table S2). Additionally, βV1 likely possesses nucleoside/nucleotide-binding or catalytic or ion channel activity (Supplementary Table S2). Homeostasis of positively charged ions such as potassium (K^+^) and calcium Ca^2+^ in the cell is crucial for the maintaining cell fate. Furthermore, Ca^2+^ ions influx plays a significant role in elicitor perception that primes major signaling cascades during host-pathogen interaction (Garcia-Brugger et al., 2006). Although the experimental evidence is lacking, there is a possibility that βV1 may regulate anion transporter which, upon activation by protein kinases aids in the generation of reactive oxygen species and mitogen-activated protein kinase (MAPK) cascade signaling pathways for defense (Garcia-Brugger et al., 2006). Although βC1 protein negatively interferes with the MAPK defense pathway, both βV1 and βC1 protein may act synergistically for regulating the host proteome to be functional towards severe pathogenesis (Hu et al., 2019, 2020).

The present study has elucidated the multifunctional role of a novel βV1 protein. We determined the role of βV1 as a protein elicitor and reported its dynamic localization in the presence and absence of viral AC3 protein. The interaction studies have also paved the way to decipher the additional biological significance of βV1 protein in viral pathogenesis.

## Data availability statement

The raw data supporting the conclusions of this article will be made available by the authors, without undue reservation.

## Author contributions

KR, NG, and SC conceived the idea. SC and HP supervised the experiments, revised the original draft, and helped in funding acquisition. NG, KR, PG, and YZ performed the experiments and data analysis. NG and KR wrote the original manuscript. All authors contributed to the article and approved the submitted version.

## Funding

This study was sponsored by an institutional grant (DBT-BUILDER of SLS) received by SC.

## Conflict of interest

The authors declare that the research was conducted in the absence of any commercial or financial relationships that could be construed as a potential conflict of interest.

## Publisher’s note

All claims expressed in this article are solely those of the authors and do not necessarily represent those of their affiliated organizations, or those of the publisher, the editors and the reviewers. Any product that may be evaluated in this article, or claim that may be made by its manufacturer, is not guaranteed or endorsed by the publisher.

## Footnotes

^1^https://wolfpsort.hgc.jp/ (Accessed August 12, 2022).
