# Supplementary material for: Functional characterization of a new ORF βV1 encoded by radish leaf curl betasatellite
Source: Front Plant Sci. 2022 Sep 20;13:972386. doi: 10.3389/fpls.2022.972386 (PMC9546537; doi:10.3389/fpls.2022.972386)
Supplement: Supplementary file 1 [file Table_1.docx]

**Supplementary Table S1** List and sequences of primers used in this study

| **S.No.** | **Primer** | **Sequence** |
| --- | --- | --- |
| 1 | 106KV1FP | ATCGATATGTCTATTACAGGAGCCTCTTC |
| 2 | 106KV1RP | GTCGACTTAATGAGTGTTCATCATATATG |
| 3 | RK1301FP | CCATGGATATGTCTATTACAGGAGCCTCTTCC |
| 4 | RK1301RP | ACTAGTATGAGTGTTCATCATATATGAACACT |
| 5 | RLBETA106FP | ATCGATATGACGATCAAATACAAAAACCAG |
| 6 | RLBETA106RP | GTCGACTTATACAGATGAACGCGTATACA |
| 7 | 35SBETAV1FP | ggatccATGTCTATTACAGGAGCCTCTTCC |
| 8 | 35SBETAV1RP | gtcgacTTAATGAGTGTTCATCATATATGAA |
| 9 | 5’RACE 191 GSP | GATTACGCCAAGCTTCCGACGAGTTTTAGGAGAGAGAAATTC |
| 10 | NAAC3BIFCFP | CACCATGATCACGGATTCACGCACAGGG |
| 11 | NAAC3BIFCRP | ATAAATATCGAGTTTTATATCATATGAAG |
| 12 | RLBETAV1BIFP | CACCATGTCTATTACAGGAGCCTCTTC |
| 13 | RLBETAV1BIRP | ATGAGTGTTCATCATATATGAACAC |
| 14 | KV1KT7FP | GGATCCTAATGTCTATTACAGGAGCCTCTTC |
| 15 | NAAC1FP | CCCGGGTATGGCTTCGCCACGTCGTTTTAG |
| 16 | NAAC1RP | GGATCCTCAACTCGCCTCCTGCGAATGCTCTTC |
| 17 | NAAC3FP | CATATGATGATCACGGATTCACGCACAGGGG |
| 18 | NAAC3RP | GGATCCTTAATAAATATCCAGTTTTATATC |
| 19 | NAAV1FP | GAATTCATGGCGAAGCGACCAGCAGATATCA |
| 20 | NAAV1RP | GGATCCTTAATTTGTGGCCGAATCATAAA |
| 21 | NAAV3FP | CATATGATGAAATTCACGCTACATGGCCTAT |
| 22 | NAAV3RP | GAATTCTCATCGGCCTGTTGGTCCAG |
| 23 | GORRTRP | TCCAGCAAATTCAAGTGCAA |
| 24 | NPR1RTFP | TAGCGTTCCTTGCTGAGGTT |
| 25 | NPR1RTRP | CTCTCGCATGCTTTACCACA |
| 26 | PR1RTFP | CCTCGTACATTCTCATGGTCAAT |
| 27 | PR1RTRP | CCATTGTTACACTGAACCCTAGC |
| 28 | PDF1.2RTFP | TGGCAAAATCTATGCGCTTT |
| 29 | PDF1.2RTRP | ATCCTTCGGTCAAACAGACG |
| 30 | RBOHBRTFP | TTTTCTCTGAGGTTTGCCAGCCACCA |
| 31 | RBOHBRTRP | GCCTTCATGTTGTTGACAATGTCTTT |
