# Supplementary material for: Functional characterization of a new ORF βV1 encoded by radish leaf curl betasatellite
Source: Front Plant Sci. 2022 Sep 20;13:972386. doi: 10.3389/fpls.2022.972386 (PMC9546537; doi:10.3389/fpls.2022.972386)
Supplement: Supplementary file 2 [file Table_2.DOCX]

**Supplementary Table S2** Predicted molecular functions and localization of βV1 protein

| **GO term** | **Name** | **Probability** | **SVM Reliability** |
| --- | --- | --- | --- |
| **Biological process predictions** | | | |
| GO:0009117 | nucleotide metabolic process | 0.919 | High |
| GO:0006810 | transport | 0.870 | High |
| GO:0019222 | regulation of metabolic process | 0.852 | High |
| GO:0016310 | phosphorylation | 0.851 | High |
| GO:0019637 | organophosphate metabolic process | 0.847 | High |
| GO:0034220 | ion transmembrane transport | 0.843 | High |
| GO:0015992 | proton transport | 0.841 | High |
| GO:0006812 | cation transport | 0.825 | High |
| GO:0098655 | cation transmembrane transport | 0.818 | High |
| GO:0015980 | energy derivation by oxidation of organic compounds | 0.818 | High |
| **Molecular Function Predictions** | | | |
| GO:0032549 | ribonucleoside binding | 0.965 | High |
| GO:0016301 | kinase activity | 0.941 | High |
| GO:0001882 | nucleoside binding | 0.940 | High |
| GO:0003824 | catalytic activity | 0.925 | High |
| GO:0035639 | purine ribonucleoside triphosphate binding | 0.924 | High |
| GO:0001883 | purine nucleoside binding | 0.924 | High |
| GO:0030554 | adenyl nucleotide binding | 0.919 | High |
| GO:0022890 | inorganic cation transmembrane transporter activity | 0.914 | High |
| GO:0000166 | nucleotide binding | 0.910 | High |
| GO:0016746 | transferase activity, transferring acyl groups | 0.904 | High |
| GO:0017076 | purine nucleotide binding | 0.902 | High |
| **Cellular component predictions** | | | |
| GO:0016021 | integral component of membrane | 0.998 | High |
| GO:0031224 | intrinsic component of membrane | 0.990 | High |
| GO:0016020 | membrane | 0.973 | High |
| GO:0031090 | organelle membrane | 0.834 | High |
| GO:0005743 | mitochondrial inner membrane | 0.821 | High |
| GO:0005886 | plasma membrane | 0.820 | High |
| GO:0005740 | mitochondrial envelope | 0.796 | High |
| GO:0019866 | organelle inner membrane | 0.775 | High |
| GO:0071944 | cell periphery | 0.761 | High |
| GO:0042175 | nuclear outer membrane-endoplasmic reticulum membrane network | 0.757 | High |
