# Supplementary material for: Functional characterization of a new ORF βV1 encoded by radish leaf curl betasatellite
Source: Front Plant Sci. 2022 Sep 20;13:972386. doi: 10.3389/fpls.2022.972386 (PMC9546537; doi:10.3389/fpls.2022.972386)
Supplement: Supplementary file 3 [file Data_Sheet_1.pdf]

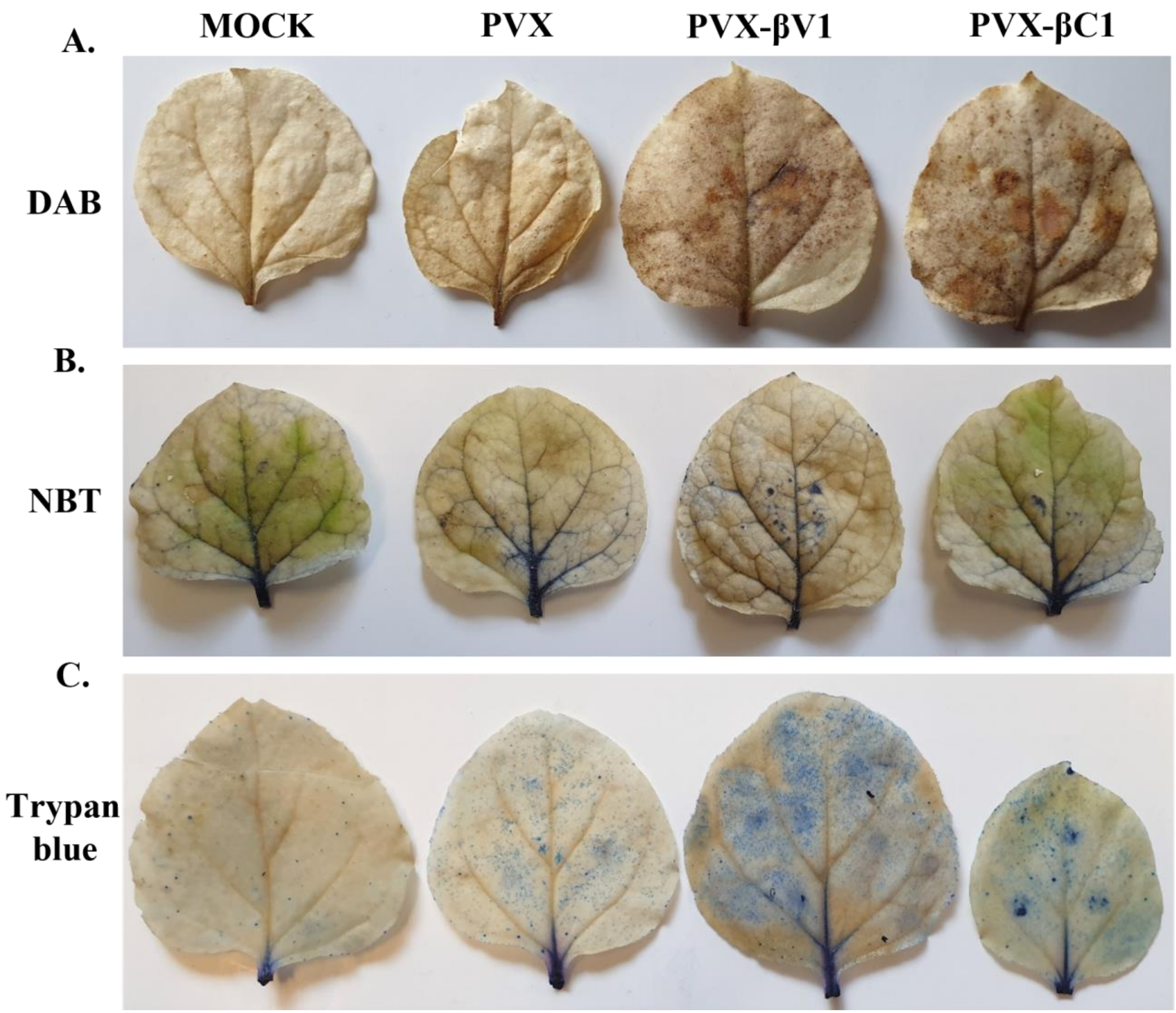

Supplementary Figure S1 Photographed PVX constructs infiltrated leaf pictures stained with DAB, NBT and trypan blue (at 5 dpi).

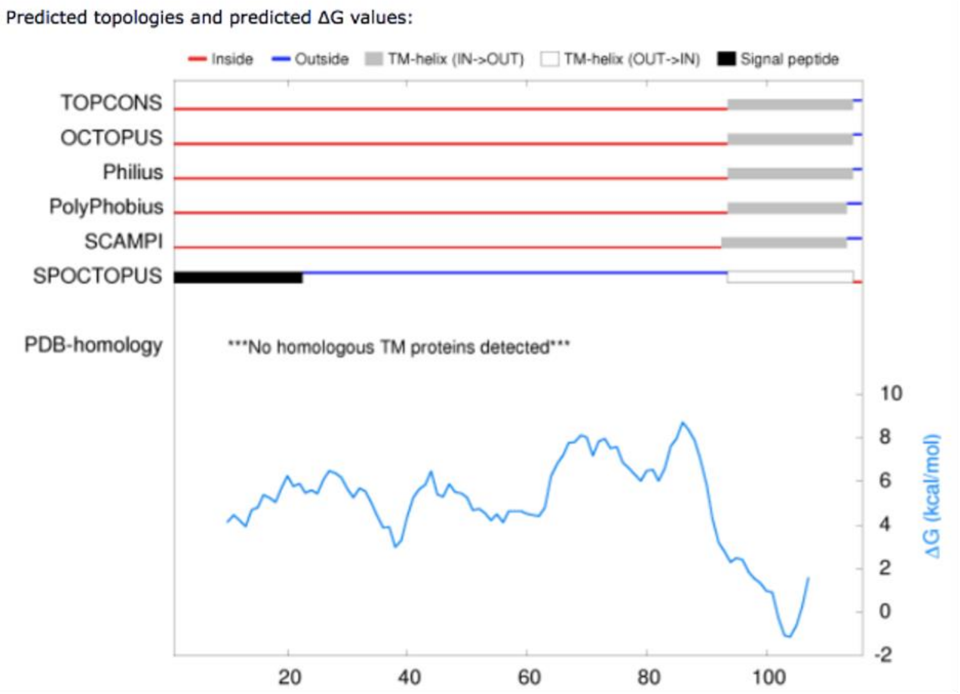

Predicted signal peptide and TM-helix positions (position starting from 1):

TOPCONS	**TM1:** 94-114	
OCTOPUS	**TM1:** 94-114	
Philius	**TM1:** 94-114	
PolyPhobius	**TM1:** 94-113	
SCAMPI	**TM1:** 93-113	
SPOCTOPUS	**SP:** 1-23,	**TM1:** 94-114
PDB-homology	***No homologous TM proteins detected***	

Supplementary Figure S2 βV1 protein topology prediction. Transmembrane topology derived using TOPCONS web server, the consensus prediction of transmembrane domain by individual sub-method are represented and predicted G values are indicated in the graph
